# Interpretable Machine Learning Framework for Predicting Major Adverse Cardiovascular Events in Rheumatoid Arthritis Using Electronic Health Records: Multicenter Cohort Study

**DOI:** 10.2196/91790

**Published:** 2026-06-05

**Authors:** Wei-Chen Chiang, Guan-Ling Lin, Yu-Sheng Chang, Yu-Chen Liu, Chih-Wei Huang, Phung Anh Nguyen, Jason C Hsu, Der-Ming Liou, Hsuan-Chia Yang

**Affiliations:** 1 Graduate Institute of Biomedical Informatics, College of Medical Science and Technology, Taipei Medical University, 9F, Education and Research Building, Shuang Ho Campus No. 301, Yuantong Rd, Zhonghe Dist, New Taipei, 235, Taiwan, 886 2-6620-2589 ext 10927; 2 Division of Allergy, Immunology and Rheumatology, Department of Internal Medicine, Taipei Medical University-Shuang Ho Hospital, Ministry of Health and Welfare, New Taipei City, Taiwan; 3 Division of Allergy, Immunology and Rheumatology, Department of Internal Medicine, School of Medicine, College of Medicine, Taipei Medical University, Ministry of Health and Welfare, Taipei, Taiwan; 4 School of Nursing, College of Medicine, National Taiwan University, Taipei, Taiwan; 5 International Center for Health Information and Technology, College of Medical Science and Technology, Taipei Medical University, Taipei, Taiwan; 6 Graduate Institute of Data Science, College of Management, Taipei Medical University, Taipei, Taiwan; 7 Clinical Big Data Research Center, Taipei Medical University Hospital, Taipei, Taiwan; 8 Research Center of Healthcare Industry Data Science, College of Management, Taipei Medical University, Taipei, Taiwan; 9 Clinical Data Center, Office of Data Science, Taipei Medical University, Taipei, Taiwan; 10 International PhD Program in Biotech and Healthcare Management, College of Management, Taipei Medical University, Taipei, Taiwan; 11 Master Program in Medical Informatics, National Tsing Hua University, Hsinchu, Taiwan; 12 Research Center of Big Data and Meta-Analysis, Wanfang Hospital, Taipei Medical University, Taipei, Taiwan

**Keywords:** major adverse cardiovascular events, MACEs, rheumatoid arthritis, random survival forest, DeepSurv, Cox-Time, Shapley additive explanations, SHAP, interpretable machine learning

## Abstract

**Background:**

Patients with rheumatoid arthritis (RA) face higher risks of major adverse cardiovascular events than the general population. While machine learning offers powerful predictive capabilities, its clinical adoption is hindered by the “black-box” nature of complex algorithms.

**Objective:**

This study aimed to develop interpretable survival models to predict the risk of major adverse cardiovascular events in patients with RA, providing transparent and actionable insights for personalized clinical prognosis management.

**Methods:**

Using data from the Taipei Medical University Clinical Research Database (2011-2022) for 2461 patients with RA, machine learning survival models, including random survival forest (RSF), DeepSurv, and Cox-Time, were compared with the traditional Cox proportional hazards model. Performance was evaluated using the C-index and integrated Brier score. Permutation importance and Shapley additive explanations (SHAP) analyses were integrated to provide explainability for individual-level risk predictions.

**Results:**

RSF demonstrated superior performance, achieving a C-index of 0.8771 and an integrated Brier score of 0.0775. Permutation importance identified key features, including creatinine, conventional synthetic disease-modifying antirheumatic drugs, C-reactive protein, alanine aminotransferase, and age at RA diagnosis. SHAP analysis further quantified feature-specific effects, revealing both protective and risk-increasing associations between medications and laboratory indicators.

**Conclusions:**

RSF outperformed traditional methods, and integrating SHAP enabled transparent, personalized risk interpretation, translating complex models into actionable insights for clinicians. This approach empowers clinicians to identify high-risk individuals and advances precision medicine in rheumatology. Future work should include temporal validation using data from later years and external validation using datasets from other health care systems to further assess model generalizability.

## Introduction

Rheumatoid arthritis (RA) is a prevalent autoimmune disease characterized by chronic synovial inflammation leading to pain, swelling, and functional disability. Beyond articular manifestations, the systemic inflammation associated with RA, together with potential treatment-related adverse effects, increases the risk of extra-articular complications such as cardiovascular disease, pulmonary disease, and malignancy [1]. Compared with the general population, patients with RA have an approximately 55% higher risk of cardiovascular events [2] and 40% higher cardiovascular disease–related mortality [3], underscoring the need for improved risk stratification and management.

Clinically, comprehensive risk evaluation is time-consuming, requiring clinicians to manually integrate numerous clinical features from medical records. Despite this demand, research specifically focused on developing cardiovascular risk prediction systems tailored for patients with RA remains scarce. Although a few assessment tools have been established, external validation reveals that they frequently underestimate risk, particularly in high-risk individuals and active smokers [4].

Several studies have applied traditional survival analysis to predict cardiovascular outcomes in RA populations [5-10]. Although these models incorporate time-to-event information and account for censored observations, they have several limitations. These models often rely on assumptions such as proportional hazards (PH) and linear relationships between covariates and risk [11,12] and may have difficulty modeling complex interactions, nonlinear effects, and high-dimensional features [13,14].

Machine learning (ML) and deep learning (DL) have emerged as powerful tools in medical research, playing key roles in diagnosis, prognosis, treatment planning, and medical image analysis, with strong performance in predicting adverse outcomes [15-17]. In survival analysis, advanced time-to-event ML and DL models, including random survival forest (RSF) [18], DeepSurv [19], and Cox-Time [20], offer superior flexibility over traditional methods by bypassing rigid PH and linearity assumptions. By autonomously capturing complex nonlinear relationships and high-order interactions in high-dimensional data, these models provide more precise and personalized risk predictions [15,16,21,22]. Despite their demonstrated success in clinical outcome prediction, their application to major adverse cardiovascular events (MACEs) in RA, particularly among Asian populations, remains underexplored. In addition, the widespread clinical implementation of these high-performing models faces challenges primarily due to their limited interpretability. The “black-box” nature of many algorithms limits transparency, making the prediction process and underlying reasoning difficult to understand, thereby reducing clinical trust.

This study aimed to adopt advanced ML and DL approaches to evaluate the risk of MACEs among patients with RA and incorporate the Shapley additive explanations (SHAP) framework [23] to develop and validate an interpretable time-to-event prediction model. By doing so, it demonstrates the feasibility and accuracy of ML-based survival models in MACE prediction, thereby providing case managers with a practical tool for individualized risk assessment and supporting personalized disease management.

## Methods

### Study Framework

Clinical data were retrospectively obtained from the Taipei Medical University Clinical Research Database (TMUCRD) in a deidentified format to ensure patient privacy. This database integrates electronic medical records from 3 affiliated hospitals within the Taipei Medical University system: Taipei Medical University Hospital, Taipei Municipal Wanfang Hospital, and Shuang Ho Hospital (managed by the Ministry of Health and Welfare, Taiwan). The TMUCRD provides comprehensive longitudinal data, including structured clinical information (demographics, diagnostic records, pharmacological treatments, and laboratory results) and unstructured narratives (physician progress notes, radiology and pathology reports, and discharge summaries), which encompass clinical features that support risk prediction modeling [24]. Data spanning 2011 to 2022 were analyzed. The study framework consisted of the following phases: cohort identification, feature engineering, model development (including training, evaluation, and interpretation), and risk prediction.

### Cohort Identification and Outcome Definition

Patients aged 18 years or older with newly diagnosed RA were identified from the TMUCRD using *International Classification of Diseases, Ninth Revision, Clinical Modification* (*ICD-9-CM*), codes 714.0 to 714.2 and *International Classification of Diseases, Tenth Revision, Clinical Modification* (*ICD-10-CM*), codes M05 to M06. To ensure the inclusion of incident RA cases, we applied a minimum 12-month washout period prior to the first RA diagnosis, requiring at least 12 months of RA-free observation after database entry. Individuals with fewer than 3 rheumatology outpatient visits were excluded to reduce diagnostic misclassification. The occurrence of subsequent incident MACEs during follow-up was identified using *ICD-9-CM* and *ICD-10-CM* codes for the following events: stroke (*ICD-9-CM*: 430-437.xx; *ICD-10-CM*: G45.x, H34.1, and I60.x-I69.x), acute myocardial infarction (*ICD-9-CM*: 410.xx; *ICD-10-CM*: I21.xx and I22.xx), heart failure (*ICD-9-CM*: 428.xx; *ICD-10-CM*: I11.0, I50.xx, and I97.1), and acute coronary syndrome and ischemic heart disease (*ICD-9-CM*: 410-414.xx; *ICD-10-CM*: I21-I24.xx). If patients had any MACEs documented before the RA diagnosis, they were excluded.

### Feature Engineering

#### Feature Retrieval

Feature variables (clinical characteristics) associated with MACE occurrence were selected as candidate predictors based on a literature review and expert consultation [8,25,26]. Demographic variables, including age at RA diagnosis and sex, were defined at baseline. Other clinical variables, including comorbidities, laboratory data, and medication use, were derived from structured clinical records and incorporated into the analysis as patient-level covariates.

Comorbidities, including diabetes mellitus (DM), hypertension, hyperlipidemia, chronic obstructive pulmonary disease (COPD), cancer, and interstitial lung disease (ILD), were identified using *ICD-9-CM* and *ICD-10-CM* codes. In addition, because the concurrent presence of DM, hypertension, hyperlipidemia, and COPD may indicate a greater comorbidity burden associated with higher MACE risk, a composite variable, multiple comorbidities, was created and included in the analysis. Laboratory variables assessed in this study included rheumatoid factor (RF), erythrocyte sedimentation rate (ESR), C-reactive protein (CRP), aspartate aminotransferase (AST), alanine aminotransferase (ALT), and serum creatinine.

Medication use, including conventional synthetic disease-modifying antirheumatic drugs (csDMARDs), biologic disease-modifying antirheumatic drugs (bDMARDs), glucocorticoids, lipid-lowering agents, and antidiabetic agents, was identified using Anatomical Therapeutic Chemical codes. Variables describing treatment exposure and combination therapy patterns were constructed for analysis. Combination definitions were as follows: combination 0 represented not meeting any of the criteria; combination 1 represented concurrent use of methotrexate and glucocorticoids for more than 168 days; combination 2 represented concurrent use of methotrexate, other csDMARDs, and glucocorticoids for more than 168 days; and combination 3 represented concurrent use of methotrexate and a bDMARD for more than 168 days.

#### Data Preprocessing

To ensure data quality and minimize the potential bias associated with excessive missingness, variables with more than 50% missing values were excluded [27,28]. Missing data in the remaining variables were addressed using an iterative chained equations imputation procedure based on multiple imputation by chained equations with Bayesian ridge regression implemented via the *HyperImpute* package in Python (Python Software Foundation) [29]. In this procedure, each variable with missing values was imputed sequentially using the other variables as predictors, and the conditional models were updated in a round-robin fashion based on observed and previously imputed values. The maximum number of iterations was set to 100 to improve the stability of the imputation process. After iterative updating, a single completed dataset was generated and used for subsequent model development and evaluation.

For continuous variables, including clinical laboratory results and medication duration, *Z*-score normalization was applied to reduce potential bias arising from differences across units or scales. This transformation rescaled the variables to have a mean of 0 and an SD of 1, ensuring comparability across features and facilitating model convergence. For categorical medication combinations (4 types), one-hot encoding was applied to convert drug exposures into independent binary indicators. This approach enabled the model to distinguish treatment strategies and evaluate the effects of specific drug combinations on event risk.

### Model Development

#### Overview

To establish an optimal risk prediction model for MACEs in patients with RA, 3 ML-based time-to-event models were developed: RSF, DeepSurv, and Cox-Time. RSF, an ensemble learning algorithm similar to bagging, builds multiple survival trees by randomly selecting features and splitting nodes to maximize survival differences between child nodes [30]. DeepSurv, a DL-based algorithm, extends the Cox PH model to capture nonlinear relationships between input features and event occurrence. It consists of multiple hidden layers and is trained using techniques such as batch normalization and gradient descent optimization [19]. Cox-Time is another DL-based algorithm that models interactions between time and covariates by treating time as a standard covariate [20]. These models were selected based on their proven effectiveness in previous survival analysis studies [31-33]. RSF was chosen for its ability to model complex, nonlinear interactions without strong parametric assumptions; DeepSurv was chosen for its flexibility in learning deep feature representations; and Cox-Time was chosen for its explicit relaxation of the PH assumption.

#### Model Training

Subsequently, the dataset was stratified by MACE occurrence and then randomly divided into an 80% training set and a 20% test set [34]. Stratified splitting ensured that the proportions of events and nonevents in both subsets were consistent with those in the original dataset, thereby enhancing the generalizability of model training and evaluation. All models were trained and evaluated using the same 80% training dataset and identical data splits to ensure fair comparison.

#### Model Evaluation and Optimization

Model performance was evaluated using the Harrell concordance index (C-index), which quantifies the model’s ability to rank the order of events correctly [29]. The integrated Brier score (IBS) was additionally used to assess the overall accuracy of predicted survival probabilities and the calibration of the model [35]. Previous studies have reported C-index values ranging between 0.7 and 0.8, indicating moderate to good discriminative ability [31,33,36]. In practical applications, models with a Brier score below 0.25 are generally considered to have acceptable predictive performance [31].

Model performance was optimized through hyperparameter tuning using GridSearchCV with 5-fold cross-validation on the 80% training set. The training data were partitioned into 5 folds, with *K* – 1 folds used for model training and the remaining fold used for validation. This procedure was repeated *K* times, and the mean C-index across validation folds was computed to determine the optimal hyperparameter configuration. Each model was subsequently retrained on the full 80% training set using the selected hyperparameters and evaluated on the independent 20% test set.

#### Model Interpretability

Model interpretability was examined using 2 complementary approaches. First, permutation importance was applied to identify features that had a substantial impact on model prediction accuracy [37,38]. This method was evaluated separately on the training and testing sets, with each feature randomly permuted 15 times to reduce the uncertainty associated with a single permutation. The average change in the C-index resulting from these permutations was used to quantify the importance of each variable. Second, the SHAP method was used to interpret individual predictions, illustrating both the direction and magnitude of feature effects and visualizing the relationships between feature values and predicted MACE risk [23].

### Conventional Cox PH Regression

To provide a benchmark for comparison with ML-based survival models, a conventional Cox PH regression approach was used. The patient cohort was stratified according to MACE occurrence. Univariable and multivariable Cox PH regression analyses were performed to evaluate the associations between clinical features and MACE risk. Kaplan-Meier (KM) analysis was further conducted to illustrate the cumulative incidence of MACEs over time, and log-rank tests were applied to compare risk differences between groups [25].

### Statistical Analysis

Continuous variables were summarized as means and SDs, whereas categorical variables were presented as counts and percentages. Differences in clinical characteristics were assessed using chi-square tests for categorical variables and 2-tailed independent-sample *t* tests for continuous variables, with statistical significance defined as a *P* value of less than .05. All statistical analyses and computations were performed using Python (version 3.11.4) and SAS (version 9.4; SAS Institute Inc).

### Ethical Considerations

This study was approved by the Taipei Medical University Joint Institutional Review Board (approval number N202312006) and authorized by the Data Management Center and the Institutional Data Governance Committee of Taipei Medical University.

## Results

### Cohort Identification and Dataset Allocation

**Figure 1.**
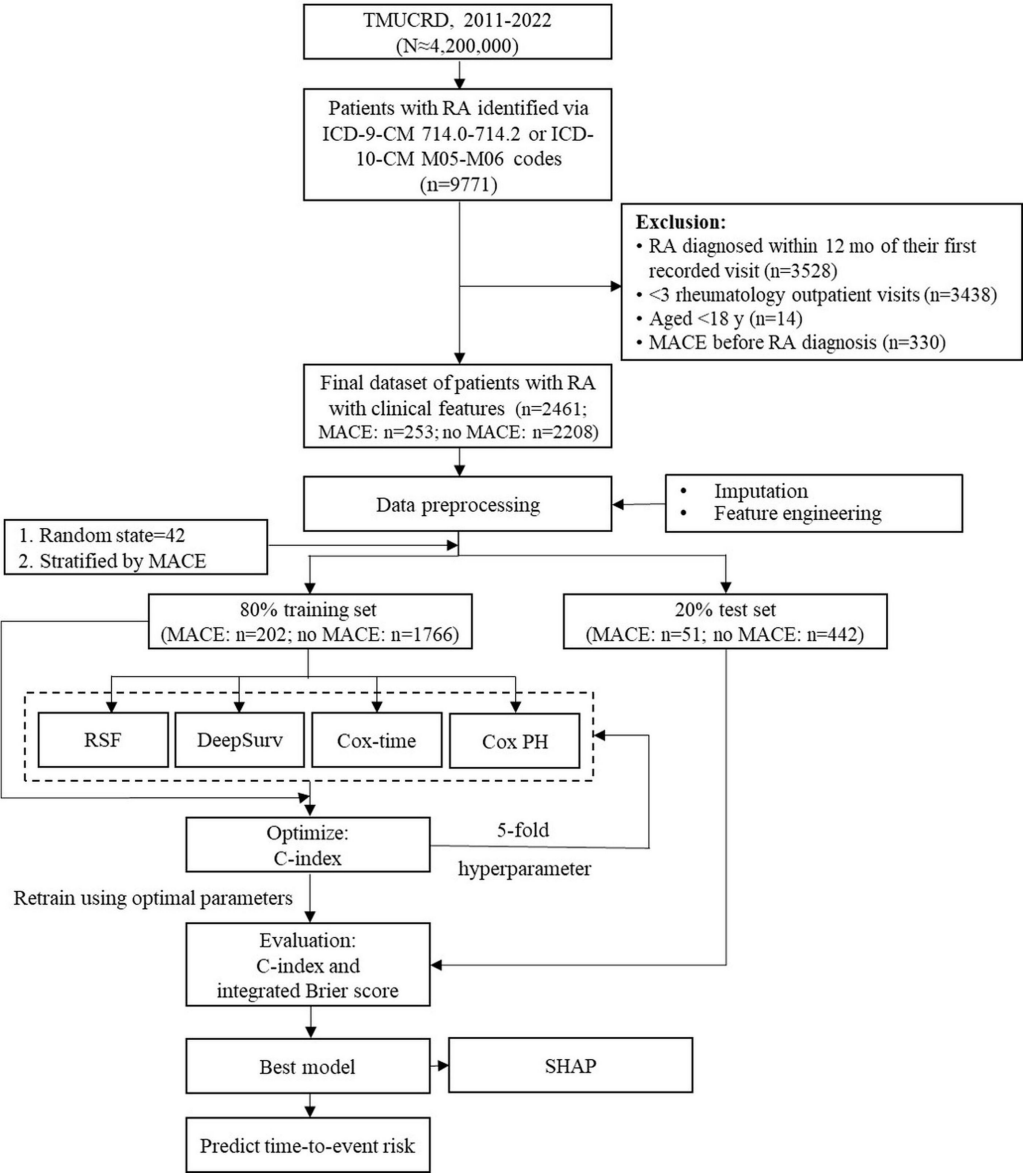
Enrollment and study workflow. *ICD-9-CM*: *International Classification of Diseases, Ninth Revision, Clinical Modification*; *ICD-10-CM*: *International Classification of Diseases, Tenth Revision, Clinical Modification*; MACE: major adverse cardiovascular event; PH: proportional hazards; RA: rheumatoid arthritis; RSF: random survival forest; SHAP: Shapley additive explanations; TMUCRD: Taipei Medical University Clinical Research Database.

Figure 1 shows the cohort identification and study workflow. From approximately 4.2 million individuals in the TMUCRD, 9771 patients with RA were initially identified. After applying the exclusion criteria, 2461 patients were included in the final analysis, of whom 253 (10.3%) experienced MACEs. The overall mean age was 51.9 (SD 13.9) years, and most patients were female (1917/2461, 77.9%). The dataset was stratified according to MACE occurrence and randomly divided into an 80% training set and a 20% test set. No significant differences in variable distributions were observed between the 2 subsets, ensuring their comparability for model development and evaluation (Table 1).

**Table 1.** Patient characteristics in the overall cohort, training set, and test set (N=2461).

	Overall	Training (n=1968)	Test (n=493)	*P* value
Experienced MACEs^a^, n (%)	253 (10.3)	202 (10.3)	51 (10.3)	—^b^
Follow-up (mo), mean (SD)	44 (32.4)	44 (32.3)	43.8 (32.8)	.92
Demographics				
Sex (female), n (%)	1917 (77.9)	1531 (77.8)	386 (78.3)	.86
Age (y), mean (SD)	51.9 (13.9)	52.2 (13.8)	51.1 (14.4)	.13
Comorbidities, n (%)				
Diabetes mellitus	246 (10.0)	200 (10.2)	46 (9.3)	.64
Hypertension	483 (19.6)	388 (19.7)	95 (19.3)	.87
Hyperlipidemia	564 (22.9)	464 (23.6)	100 (20.3)	.14
COPD^c^	321 (13.0)	255 (13.0)	66 (13.4)	.86
MC^d^	19 (0.8)	14 (0.7)	5 (1.0)	.56
ILD^e^	17 (0.7)	10 (0.5)	7 (1.4)	.06
Cancer	106 (4.3)	88 (4.5)	18 (3.7)	.50
Laboratory tests, mean (SD)				
Rheumatoid factor, IU/mL	80.9 (251.0)	81.8 (215.2)	77.4 (360.1)	.79
C-reactive protein, mg/dL	0.9 (2.9)	0.9 (3.0)	0.7 (2.4)	.22
ESR^f^, mm/h	17.2 (18.5)	17.5 (18.8)	15.8 (17.3)	.06
AST^g^, U/L	24.4 (26.9)	24.2 (28.3)	24.9 (20.4)	.57
ALT^h^, U/L	23.6 (30.2)	23.3 (29.4)	24.8 (33.1)	.37
Creatinine, mg/dL	0.9 (2.3)	0.9 (2.5)	0.9 (0.8)	.54
Medication use (d), mean (SD)				
bDMARDs^i^	136.9 (456.4)	135.4 (459.2)	142.6 (445.4)	.75
csDMARDs^j^	1037.6 (1461.5)	1034.2 (1462.5)	1051.2 (1459.1)	.82
Glucocorticoids	302.5 (501.0)	307.5 (505.4)	282.7 (483.0)	.31
Lipid-lowering agents	97.2 (361.5)	95.9 (339.9)	102.4 (437.7)	.76
Antidiabetic agents	45 (289.5)	43.9 (285.5)	49.5 (305.4)	.71
Medication combination^k^, n (%)				.33
Combination 0	1800 (73.1)	1436 (73.0)	364 (73.8)	
Combination 1	58 (2.4)	50 (2.5)	8 (1.6)	
Combination 2	569 (23.1)	458 (23.3)	111 (22.5)	
Combination 3	34 (1.4)	24 (1.2)	10 (2.0)	

a MACE: major adverse cardiovascular event.

b Not available.

c COPD: chronic obstructive pulmonary disease.

d MC: multiple comorbidities, defined as concurrent diabetes, hypertension, hyperlipidemia, and COPD.

e ILD: interstitial lung disease.

f ESR: erythrocyte sedimentation rate.

g AST: aspartate aminotransferase.

h ALT: alanine aminotransferase.

i bDMARD: biologic disease-modifying antirheumatic drug.

j csDMARD: conventional synthetic disease-modifying antirheumatic drug.

k Combination definitions: combination 0 encompasses not meeting any of the criteria; combination 1 encompasses concurrent use of methotrexate and glucocorticoids for more than 168 days; combination 2 encompasses concurrent use of methotrexate, other csDMARDs, and glucocorticoids for more than 168 days; and combination 3 encompasses concurrent use of methotrexate and a bDMARD for more than 168 days.

### Model Performance and Interpretability

For each model, candidate hyperparameters were tuned using 5-fold cross-validation with GridSearchCV on the training set. The configuration yielding the highest average C-index across validation folds was retained as the optimal setting, as summarized in Table 2. Each model was subsequently retrained on the full training set using these optimized hyperparameters and evaluated on the independent test set, with detailed results shown in Table 3. Among all models, RSF achieved the best performance during 5-fold cross-validation, with an average C-index of 0.8443 (SD 0.0373; 95% CI 0.8116-0.8771). Consistent with the cross-validation results, the RSF model maintained the highest performance on the independent test set (C-index=0.8771; IBS=0.0775).

**Table 2.** Model performance in 5-fold cross-validation with hyperparameter tuning via GridSearchCV.

Model	Fold	C-index^a^, mean (SD; 95% CI)	Optimal hyperparameter
RSF^b^	Fold 1: 0.8912 Fold 2: 0.8524 Fold 3: 0.8272 Fold 4: 0.7824 Fold 5: 0.8685	0.8443 (0.0373; 0.8116-0.8771)	{‘max_features’: ‘sqrt’, ‘min_samples_leaf’: 5, ‘min_samples_split’: 5, ‘n_estimators’: 200}
Cox PH^c^	Fold 1: 0.8169 Fold 2: 0.7769 Fold 3: 0.7424 Fold 4: 0.6887 Fold 5: 0.7864	0.7623 (0.0438; 0.7239-0.8006)	—^d^
Cox-Time	Fold 1: 0.8463 Fold 2: 0.8267 Fold 3: 0.7700 Fold 4: 0.7557 Fold 5: 0.7873	0.7972 (0.0342; 0.7672-0.8272)	{‘batch_norm’: True, ‘dropout’: 0.3, ‘learning_rate’: 0.001, ‘num_nodes’: [128, 128]}
DeepSurv	Fold 1: 0.8355 Fold 2: 0.8257 Fold 3: 0.8011 Fold 4: 0.7739 Fold 5: 0.8311	0.8135 (0.0231; 0.7932-0.8337)	{‘batch_norm’: True, ‘dropout’: 0.1, ‘learning_rate’: 0.001, ‘num_nodes’: [256, 256]}

a C-index: concordance index.

b RSF: random survival forest.

c PH: proportional hazards.

d Not available.

**Table 3.** Performance of the retrained model on training and test sets.

Metric and model	80% training set	20% test set
C-index^a^		
RSF^b^	0.9690	0.8771
Cox PH^c^	0.7936	0.7569
Cox-Time	0.8179	0.7989
DeepSurv	0.8896	0.8163
IBS^d^		
RSF	0.0397	0.0775
Cox PH	0.0825	0.0936
Cox-Time	0.1267	0.1298
DeepSurv	0.0632	0.0855

a C-index: concordance index.

b RSF: random survival forest.

c PH: proportional hazards.

d IBS: integrated Brier score.

**Figure 2.**
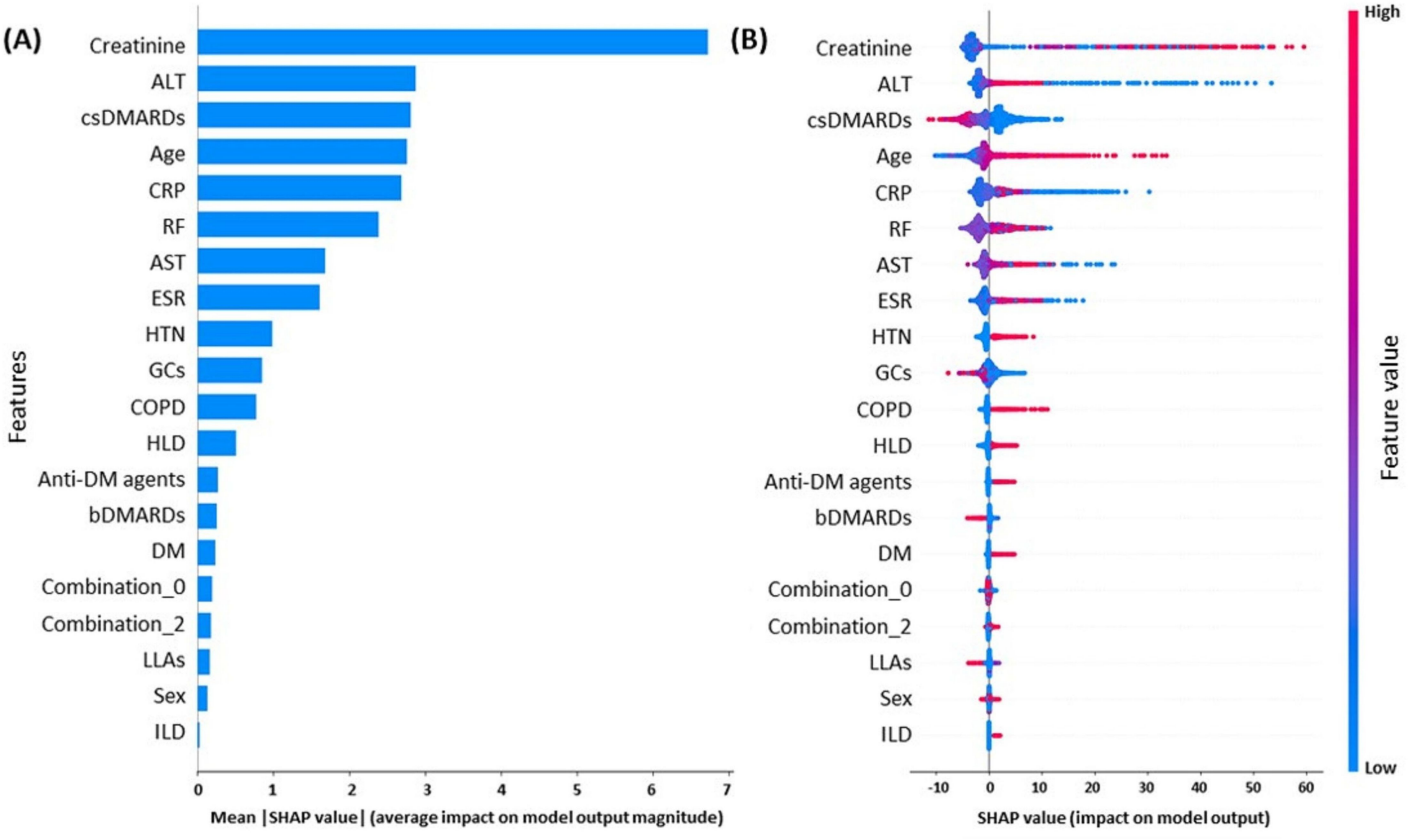
Shapley additive explanations (SHAP) summary plots for the random survival forest model. (A) Features are ranked in descending order by mean absolute SHAP value, with larger values indicating greater overall contribution to the model. (B) The SHAP summary plot shows the direction and magnitude of feature effects on predicted major adverse cardiovascular event (MACE) risk; values to the right indicate higher predicted risk, whereas values to the left indicate lower predicted risk. Red and blue dots represent higher and lower feature values, respectively. Medication combinations are defined in the Methods section and Table 1 footnote. Briefly, combination 0 indicates not meeting any medication combination criteria, and combination 2 indicates concurrent use of methotrexate, other csDMARDs, and GCs for more than 168 days. ALT: alanine aminotransferase; AST: aspartate aminotransferase; bDMARDs, biologic disease-modifying antirheumatic drugs; COPD: chronic obstructive pulmonary disease; CRP: C-reactive protein; csDMARDs: conventional synthetic disease-modifying antirheumatic drugs; DM: diabetes mellitus; ESR: erythrocyte sedimentation rate; GCs: glucocorticoids; HLD: hyperlipidemia; HTN: hypertension; ILD: interstitial lung disease; LLAs: lipid-lowering agents; RF: rheumatoid factor.

The RSF model was applied, and permutation importance was evaluated to rank feature contributions, as shown in Multimedia Appendix 1. Creatinine, csDMARDs, CRP, ALT, age, and RF were identified as the most critical features for predicting MACE risk, demonstrating markedly higher importance than other variables. Figure 2 shows the SHAP summary plots illustrating the contribution of individual features to the prediction of MACEs. Creatinine, which had the largest mean absolute SHAP values, exhibited the greatest impact on the model output, followed by ALT, csDMARDs, age, CRP, RF, AST, and ESR (left panel). In contrast, the use of csDMARDs, bDMARDs, glucocorticoids, and lipid-lowering agents showed negative SHAP values, suggesting protective factors against MACEs (right panel).

### Association Analysis and Survival Validation

For comparison with ML- and DL-based survival models, a traditional Cox PH regression was used. Multimedia Appendix 2 summarizes the clinical characteristics stratified by MACE occurrence. Patients with MACEs were older and showed a higher prevalence of hypertension, DM, hyperlipidemia, COPD, and ILD. Laboratory markers were also elevated in the MACE group, including RF, ESR, and creatinine. In contrast, patients without MACEs had longer treatment durations with bDMARDs and csDMARDs.

In the Cox PH model (Multimedia Appendix 3), univariable regression showed that an older age at RA diagnosis; comorbidities such as DM, hypertension, hyperlipidemia, COPD, and ILD; elevated ESR; and higher creatinine levels were associated with increased MACE risk . In contrast, the defined medication combination was associated with lower MACE risk. Multivariate regression further identified age at RA diagnosis, hypertension, and creatinine levels as independent predictors of MACEs. Among these, hypertension was the most prominent risk factor for MACEs.

**Figure 3.**
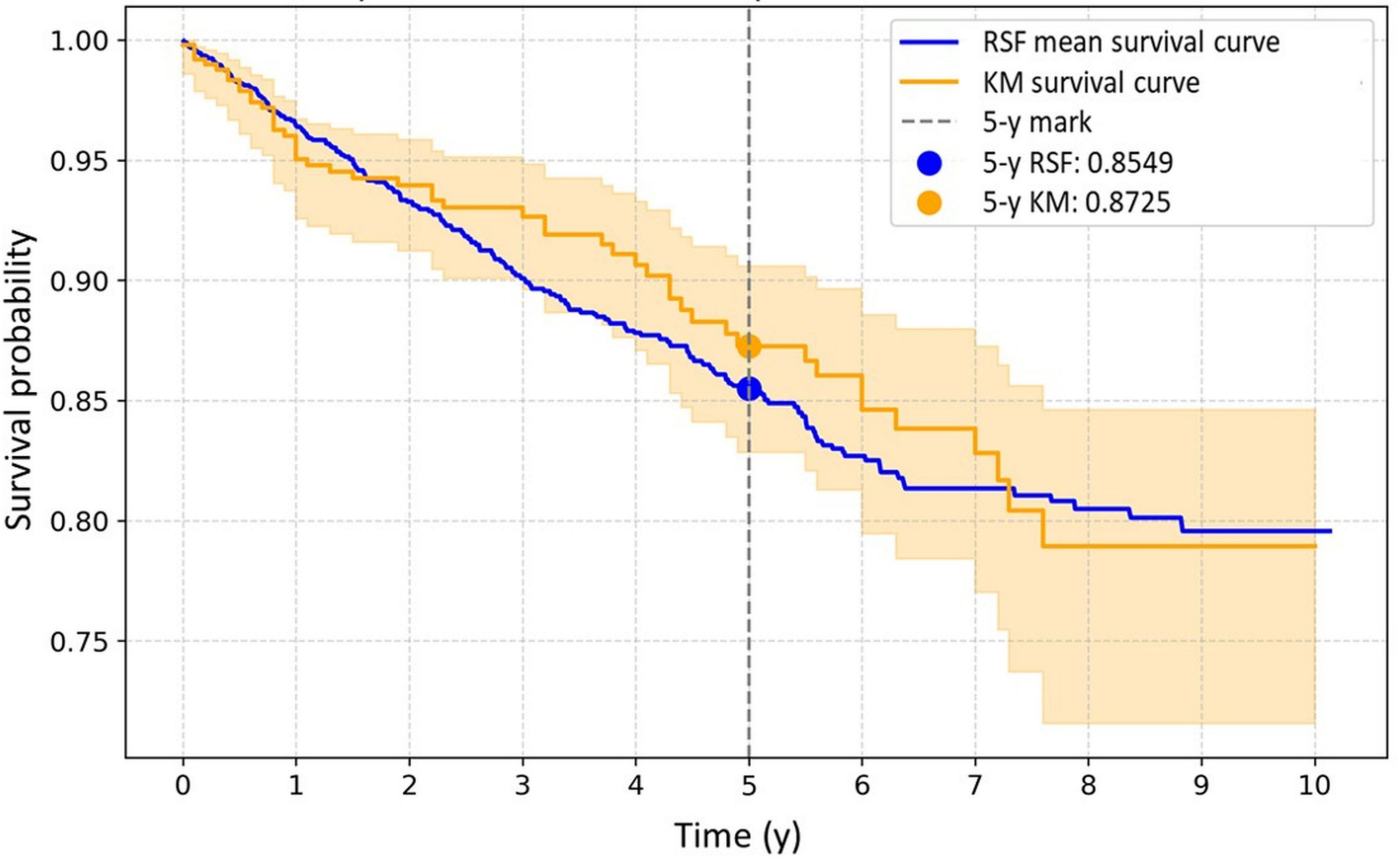
Comparison of random survival forest (RSF) and Kaplan-Meier (KM) survival curves.

For further model application and personalized interpretation, the KM survival curve for the entire cohort was overlaid with the RSF model’s average survival function based on the testing set, as shown in Figure 3, demonstrating highly consistent 5-year survival rates of 0.8725 for the KM model and 0.8549 for the RSF model.

## Discussion

### Key Findings and Contributions

To the best of our knowledge, this study is the first to use multi-hospital electronic health record (EHR) data to develop and validate interpretable time-to-event ML and DL models for predicting MACE risk in Asian patients with RA. Using real-world data from a large health care system, the RSF model achieved the best performance in the independent test set (C-index=0.8771; IBS=0.0775), outperforming the Cox PH, DeepSurv, and Cox-Time models. The strong concordance between the RSF-predicted and KM-estimated survival curves confirmed the robustness of the model. Moreover, by integrating the SHAP framework, the model provided transparent interpretability and identified key features, including creatinine, csDMARD use, CRP, ALT, and RA diagnosis age, as the most influential determinants of MACE risk in RA.

This study offers several important advances over previous prognostic research on RA. First, compared with conventional Cox models that typically rely on linear effects and PH assumptions, ML- and DL-based survival algorithms such as RSF, DeepSurv, and Cox-Time extend traditional Cox modeling by capturing nonlinear effects and, in some cases, relaxing the PH assumption. RSF and Cox-Time in particular provide more flexible modeling frameworks that may better accommodate the complexity of real-world clinical data. Second, through 5-fold cross-validation and independent test set evaluation, the RSF model achieved the best predictive performance among the models examined, supporting the feasibility of this modeling framework for MACE risk prediction in patients with RA. Third, we combined permutation importance, which quantified the overall contribution of each feature to model performance, with SHAP analysis, which enhanced individual-level interpretability, thereby linking model predictions to personalized risk assessment.

### Clinical Interpretation and Implications

Analysis of the RSF model using SHAP interpretability techniques identified creatinine, ALT, CRP, csDMARD use, and RA diagnosis age as key features associated with MACE risk. The observed associations were largely consistent with previous evidence: elevated creatinine reflects the adverse cardiovascular impact of renal dysfunction [39,40]. Higher ALT levels may reflect fatty liver disease [41], including nonalcoholic fatty liver disease and metabolic dysfunction–associated fatty liver disease, both of which have been associated with an increased risk of MACEs [42-44]. Similarly, higher CRP highlights the critical role of systemic inflammation in RA-related cardiovascular complications [45,46], and an older age at RA diagnosis has been linked to an increased risk of adverse cardiovascular outcomes [7,47]. Moreover, SHAP analysis supported prior evidence by showing that longer exposure to csDMARDs, bDMARDs [48,49], and lipid-lowering agents [50,51] aligns with reports highlighting the prognostic importance for cardiovascular outcomes of initiating effective treatment and inflammation control, as illustrated by the red-colored dots clustering toward the left (negative SHAP values) in Figure 2 (right panel).

Interestingly, previous studies have reported conflicting results regarding the association between glucocorticoids and MACEs. Cardiovascular protection appears limited to low-dose use (≤5 mg of prednisone daily) [52], whereas higher doses, prolonged use, older age, and comorbidities are associated with increased risk [53]. In SHAP analysis, glucocorticoids were linked to reduced MACE risk. Additional analysis of follow-up duration and exposure days in this study revealed that MACE cases had brief daily exposure, suggesting short-term, low-dose use. Nonetheless, MACE risk should be interpreted in relation to all clinical features, as reflected by the overall distribution of SHAP values.

In summary, our study, by integrating the RSF model with permutation importance and SHAP analyses, offers valuable insights into high-risk factors and provides interpretable predictions for clinical application. This demonstrates the feasibility of ML models for predicting MACE risk in patients with RA, potentially aiding case managers in improving patient prognosis and developing personalized treatment plans.

### Personalized Predictions Using SHAP

#### Overview

**Figure 4.**
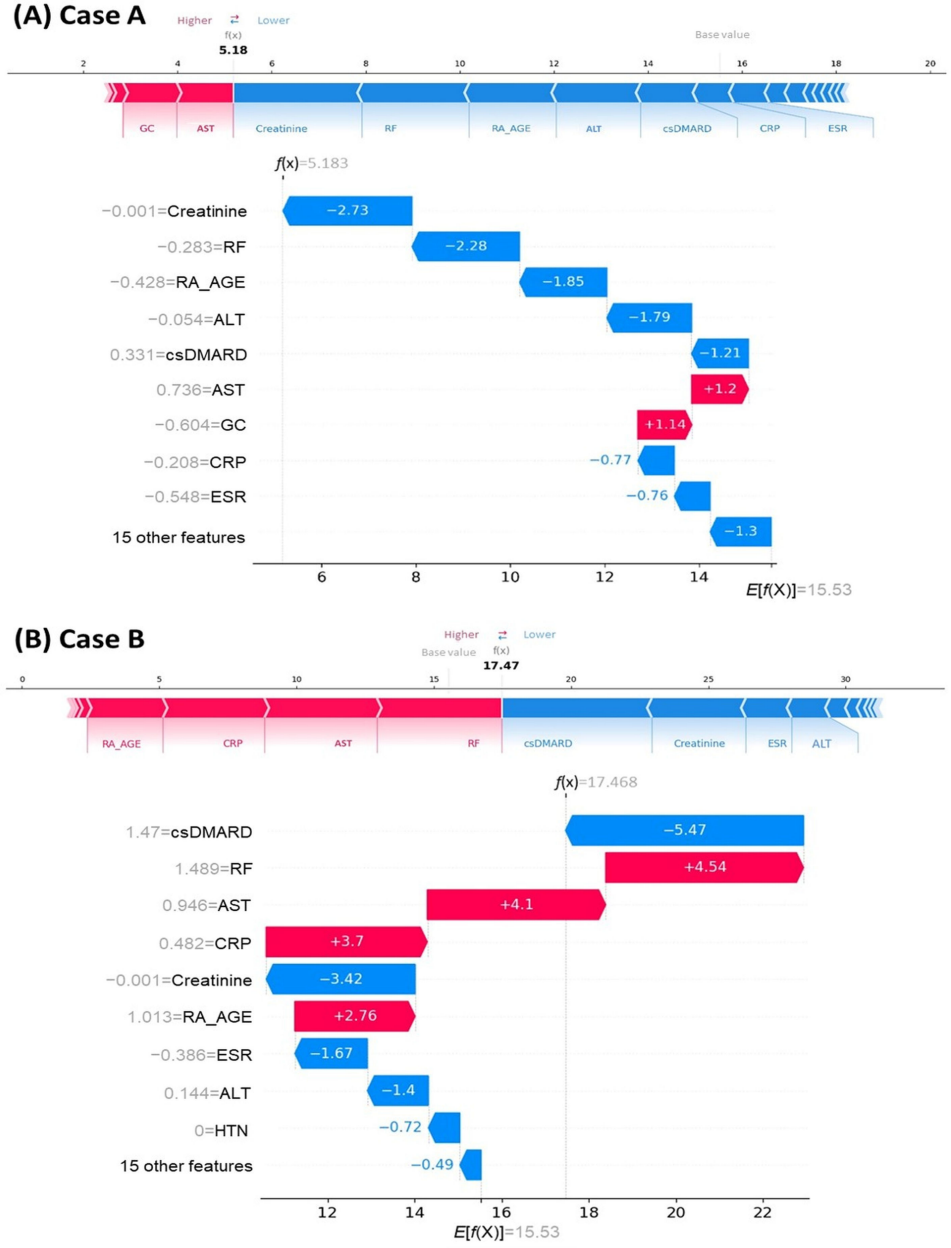
Shapley additive explanations (SHAP) values for 2 representative cases: a man aged 46 years who was major adverse cardiovascular event (MACE) free (case A) and a woman aged 66 years who experienced a MACE (case B). For each case, the top panel shows the SHAP force plot, and the bottom panel shows the waterfall plot derived from the random survival forest model. Red denotes features that increase MACE risk, whereas blue denotes features that decrease MACE risk. Arrows indicate the shift from the baseline prediction to the individualized prediction. Key contributing features include creatinine, rheumatoid factor (RF), age at rheumatoid arthritis (RA) diagnosis (RA_AGE), alanine aminotransferase (ALT) and aspartate aminotransferase (AST), conventional synthetic disease-modifying antirheumatic drug (csDMARD) use, C-reactive protein (CRP), erythrocyte sedimentation rate (ESR), glucocorticoid (GC) use, and hypertension (HTN).

SHAP values further provide granular insights into how clinical features such as age at diagnosis, sex, comorbidities, and medication use influence each patient’s risk of experiencing MACEs. To illustrate this personalized risk interpretation, we present 2 contrasting cases: a man aged 46 years who remained MACE free throughout the follow-up and a woman aged 66 years who experienced a MACE during the study. Individual SHAP force (top) and waterfall (bottom) plots for both cases are presented in Figure 4.

#### Case A: Man Aged 46 Years, MACE Free

A man aged 46 years was diagnosed with RA and followed for 54 months without MACEs. His SHAP plots indicated reduced MACE risk: starting from the baseline prediction of 15.53, the cumulative contributions of his individual features lowered the predicted risk score to 5.18 (Figure 4; case A force plot). Risk-reducing contributors included creatinine, RF, age at RA diagnosis, ALT, csDMARD use, CRP, and ESR, whereas AST and glucocorticoid use were risk increasing. Consistent with these explanations, his individual KM curve (Figure S2 in Multimedia Appendix 4, top panel) showed that the estimated time point corresponding to a MACE-free probability of 80% was 8.38 years. His 5-year MACE-free probability was 0.94, corresponding to a 5-year MACE risk of 0.06.

#### Case B: Woman Aged 66 Years, Experienced a MACE

A woman aged 66 years was diagnosed with RA and experienced a MACE after 51.1 months of follow-up. Her SHAP plots indicated elevated MACE risk: starting from the baseline prediction of 15.53, the cumulative contributions of her individual features increased the predicted risk score to 17.47 (Figure 4; case B force plot). Risk-increasing contributors included RF, AST, CRP, and age at RA diagnosis, whereas csDMARD use, creatinine, ESR, and ALT had protective effects. Consistent with these explanations, her individual KM curve (Multimedia Appendix 4, bottom panel) showed that the estimated time point at which the MACE-free probability decreased to 80% was 6.03 years. Her 5-year MACE-free probability was 0.85, equivalent to a 5-year MACE risk of 0.15.

#### Key Insights From Both Cases

Taken together, these 2 contrasting cases illustrate how the RSF-SHAP framework leverages patient-level characteristics to estimate MACE risk. The key observations are that (1) laboratory markers, including renal function (creatinine), systemic inflammation (RF, CRP, and ESR), and hepatic function (AST and ALT), were more informative than other laboratory values and contributed more to risk differentiation; (2) medication exposure (csDMARDs, bDMARDs, and glucocorticoids) highlighted the link between therapy and risk, with csDMARD exposure being the most influential treatment feature; (3) age at diagnosis was an important determinant, suggesting interactions with comorbidity burden, medication exposure, and adverse biochemical profiles; and (4) baseline comorbidities had a comparatively smaller influence than laboratory and treatment features. These patterns align with permutation importance (Multimedia Appendix 1) and the SHAP summary (Figure 2).

### Strengths and Limitations

Our study offers several significant advances over existing prognostic models. By leveraging large-scale, multi-hospital EHR data to develop survival ML models for MACE risk in patients with RA, this study demonstrates superior predictive accuracy through the RSF model’s ability to capture complex nonlinear relationships and complex feature interactions without relying on the PH assumption. By integrating SHAP analysis, it provides transparent, individual-level risk interpretations that bridge the gap between complex algorithms and personalized clinical decision-making.

Despite this study’s strengths, several limitations should be considered. First, this retrospective study involved substantial missingness in the TMUCRD dataset. Variables with more than 50% missing data were excluded as such missingness often suggests limited clinical relevance in routine care. For the remaining important variables, missing values were addressed using an iterative chained equations imputation approach informed by the multiple imputation by chained equations to preserve data structure and intervariable relationships. Although this may reduce bias compared with single imputation, excluding highly incomplete variables may still have limited our ability to capture patient heterogeneity. Missing laboratory covariates were further imputed under a missing at random assumption; however, the imputation model did not incorporate survival outcome information, which may have affected covariate-outcome associations. Second, the model was internally evaluated using a stratified train-test split and cross-validation, but neither external nor temporal validation was performed in this study. Accordingly, the generalizability of the findings to other hospitals, health care settings, ethnic populations, and calendar periods remains uncertain. In addition, calibration plots, decision curve analysis, and threshold-based performance evaluation were not included in this study. Therefore, the findings should be interpreted as preliminary rather than definitive evidence of clinical usefulness. Third, although ML-based survival models were applied, the predictors were incorporated as patient-level covariates rather than as truly time-varying covariates updated during follow-up. Therefore, the models may not fully capture dynamic changes in clinical status, laboratory results, and treatment exposure over time. Fourth, despite the use of cross-validation, the relatively modest number of events (n=253) and the complexity of the ML models may still introduce a risk of overfitting. Fifth, while disease and medication may contribute to MACE risk prediction, medication exposure in observational EHR data may be influenced by disease severity, prescribing patterns, and other potential confounders. Finally, the EHR database lacked key lifestyle covariates such as smoking status, BMI, and physical activity, which may have resulted in incomplete adjustment for potential confounding.

### Conclusions

In conclusion, this study demonstrates that the RSF model significantly outperformed traditional Cox PH and DL-based survival models in predicting MACE risk among patients with RA. By effectively capturing nonlinear relationships and feature interactions, our model provides superior predictive accuracy and robustness. The integration of permutation importance and SHAP analyses bridges the gap between complex “black-box” algorithms and clinical practice, offering a transparent framework for personalized risk assessment. These findings empower clinicians and case managers to identify high-risk individuals early and develop tailored preventive strategies. Future work should include calibration assessment, decision curve analysis, and threshold-based performance evaluation, as well as temporal validation using data from later calendar periods; external validation using data from other health care systems across diverse ethnic populations; and the incorporation of time-varying variables for clinical status, laboratory measurements, and treatment exposure to improve predictive accuracy, clinical utility, and generalizability.

## Supplementary material

10.2196/91790 Multimedia Appendix 1 Permutation importance of input features in the random survival forest model.

10.2196/91790 Multimedia Appendix 2 Clinical characteristics stratified by major adverse cardiovascular event occurrence.

10.2196/91790 Multimedia Appendix 3 Univariate and multivariate Cox proportional hazards regression analyses for predictors of major adverse cardiovascular events in patients with rheumatoid arthritis.

10.2196/91790 Multimedia Appendix 4 Kaplan-Meier survival curves for cases A and B.

## Abbreviations

ALT: alanine aminotransferase
AST: aspartate aminotransferase
bDMARD: biologic disease-modifying antirheumatic drug
COPD: chronic obstructive pulmonary disease
CRP: C-reactive protein
csDMARD: conventional synthetic disease-modifying antirheumatic drug
DL: deep learning
DM: diabetes mellitus
EHR: electronic health record
ESR: erythrocyte sedimentation rate
IBS: integrated Brier score
ICD-10-CM: International Classification of Diseases, Tenth Revision, Clinical Modification
ICD-9-CM: International Classification of Diseases, Ninth Revision, Clinical Modification
ILD: interstitial lung disease
KM: Kaplan-Meier
MACE: major adverse cardiovascular event
ML: machine learning
PH: proportional hazards
RA: rheumatoid arthritis
RF: rheumatoid factor
RSF: random survival forest
SHAP: Shapley additive explanations
TMUCRD: Taipei Medical University Clinical Research Database
