# Supplementary material for: Interpretable Machine Learning Framework for Predicting Major Adverse Cardiovascular Events in Rheumatoid Arthritis Using Electronic Health Records: Multicenter Cohort Study
Source: JMIR Form Res. 2026 Jun 5;10:e91790. doi: 10.2196/91790 (PMC13240640; doi:10.2196/91790)
Supplement: Multimedia Appendix 1 [file formative-v10-e91790-s001.docx]

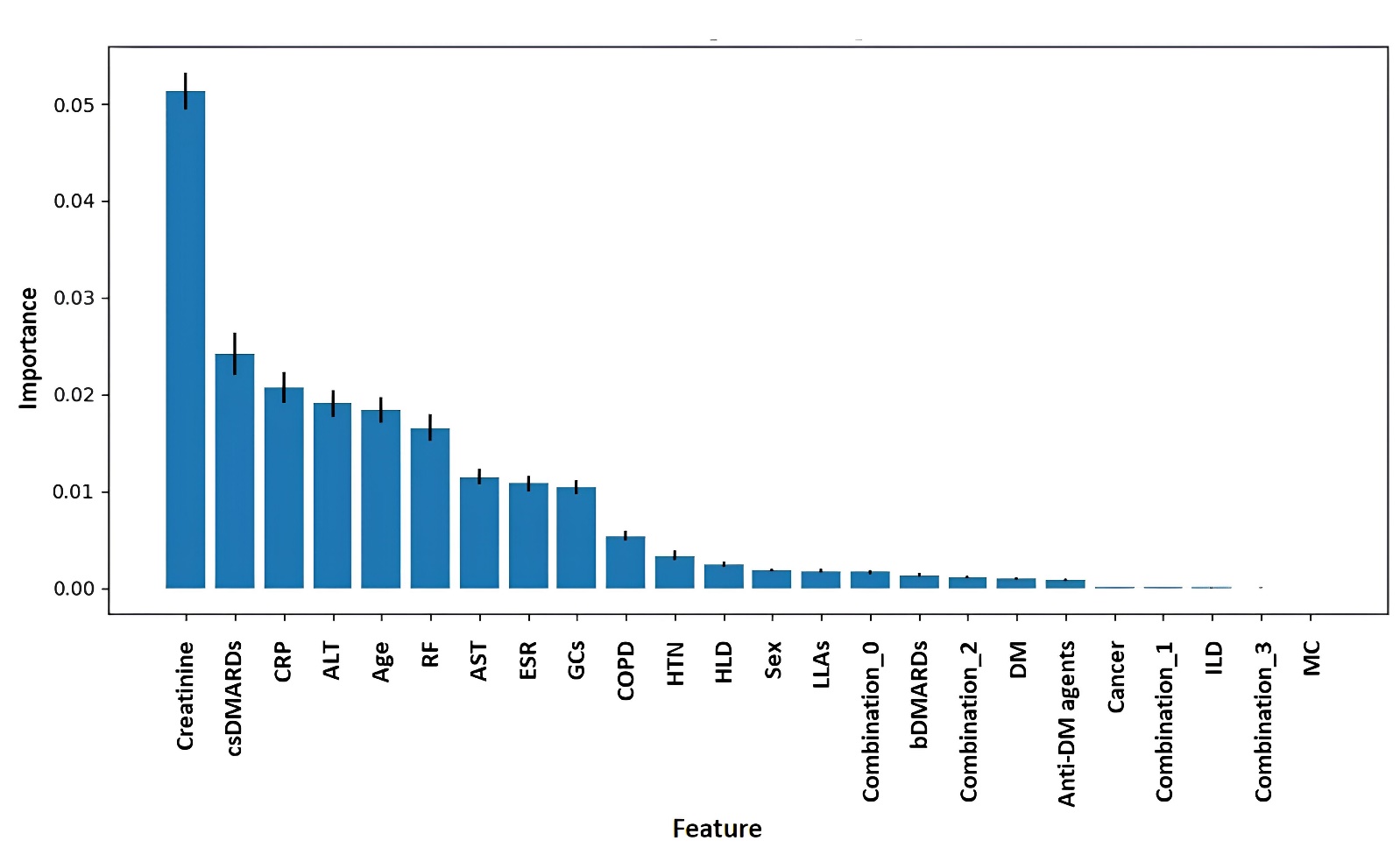


Multimedia Appendix 1. Permutation importance of input features in the random survival forest model

Note: RSF, random survival forest; CRP, C-reactive protein; ALT, alanine aminotransferase; csDMARDs, conventional synthetic disease-modifying antirheumatic drugs; Age, age at RA diagnosis; RF, rheumatoid factor; AST, aspartate aminotransferase; bDMARDs, biologic disease-modifying antirheumatic drugs; GCs, glucocorticoids; LLAs, lipid-lowering agents; ILD, interstitial lung disease; MC, multiple comorbidities, defined as concurrent diabetes, hypertension, hyperlipidemia, and chronic obstructive pulmonary disease; ESR, erythrocyte sedimentation rate; COPD, chronic obstructive pulmonary disease; DM, diabetes mellitus; HTN, hypertension; HLD, hyperlipidemia;

*Combination definitions: Combination_0, not meeting any of the criteria below; Combination_1, concurrent use of methotrexate and glucocorticoid for >168 days; Combination_2, concurrent use of methotrexate, other conventional synthetic disease-modifying antirheumatic drugs, and glucocorticoid for >168 days; Combination_3, concurrent use of methotrexate and a biologic disease-modifying antirheumatic drug for >168 days.
