# Supplementary material for: Interpretable Machine Learning Framework for Predicting Major Adverse Cardiovascular Events in Rheumatoid Arthritis Using Electronic Health Records: Multicenter Cohort Study
Source: JMIR Form Res. 2026 Jun 5;10:e91790. doi: 10.2196/91790 (PMC13240640; doi:10.2196/91790)
Supplement: Multimedia Appendix 2 [file formative-v10-e91790-s002.docx]

Multimedia Appendix 2. Clinical characteristics stratified by major adverse cardiovascular event occurrence

|  |  | No MACE | | MACE | | *P value* |
| --- | --- | --- | --- | --- | --- | --- |
|  | Number of patients | 2,208 | | 253 | |  |
|  | Follow-up, months, mean (SD) | 45.6 | (32.7) | 29.4 | (24.7) | <.001 |
| Demographics | |  |  |  |  |  |
|  | Sex (female), n (%) | 1,724 | (78.1) | 193 | (76.3) | .57 |
|  | Age, mean (SD) | 51 | (13.8) | 60 | (12.3) | <.001 |
| Comorbidities, n (%) | |  |  |  |  |  |
|  | Diabetes mellitus | 200 | (9.1) | 46 | (18.2) | <.001 |
|  | Hypertension | 392 | (17.8) | 91 | (36.0) | <.001 |
|  | Hyperlipidemia | 481 | (21.8) | 83 | (32.8) | <.001 |
|  | COPD | 263 | (11.9) | 58 | (22.9) | <.001 |
|  | MC | 15 | (0.7) | 4 | (1.6) | .12 |
|  | ILD | 11 | (0.5) | 6 | (2.4) | .01 |
|  | Cancer | 92 | (4.2) | 14 | (5.5) | .40 |
| Laboratory tests, mean (SD) | |  |  |  |  |  |
|  | Rheumatoid factor | 76.5 | (247.7) | 119.2 | (275.3) | .02 |
|  | C-reactive protein | 0.9 | (3.0) | 0.8 | (2.3) | .71 |
|  | ESR | 16.8 | (18.5) | 20 | (18.9) | .01 |
|  | AST | 24.3 | (27.3) | 24.8 | (23.1) | .78 |
|  | ALT | 23.7 | (30.8) | 23.2 | (24.7) | .79 |
|  | Creatinine | 0.9 | (2.4) | 1.3 | (1.6) | .001 |
| Medications, days of use, mean (SD) | |  |  |  |  |  |
|  | bDMARDs | 147.9 | (477.0) | 40.4 | (173.0) | <.001 |
|  | csDMARDs | 1,079.3 | (1,493.3) | 673.8 | (1,083.6) | <.001 |
|  | Glucocorticoids | 311.8 | (510.1) | 221.2 | (405.3) | .001 |
|  | Lipid-lowering agents | 99.3 | (371.0) | 79.2 | (264.3) | .28 |
|  | Antidiabetic agents | 43.5 | (286.6) | 58.4 | (313.6) | .47 |
| Medication combination^a^, n (%) | |  |  |  |  |  |
|  | Combination 0 | 1,598 | (72.4) | 202 | (79.8) | .07 |
|  | Combination 1 | 55 | (2.5) | 3 | (1.2) |  |
|  | Combination 2 | 524 | (23.7) | 45 | (17.8) |  |
|  | Combination 3 | 31 | (1.4) | 3 | (1.2) |  |

Note: n, number; MACE, major adverse cardiovascular events; SD, standard deviation; COPD, chronic obstructive pulmonary disease; MC, multiple comorbidities, defined as concurrent diabetes, hypertension, hyperlipidemia, and chronic obstructive pulmonary disease; ILD, interstitial lung disease; ESR, erythrocyte sedimentation rate; AST, aspartate aminotransferase; ALT, alanine aminotransferase; bDMARDs, biologic disease-modifying antirheumatic drugs; csDMARDs, conventional synthetic disease-modifying antirheumatic drugs;

^a^Combination definitions: Combination 0, not meeting any of the criteria below; Combination 1, concurrent use of methotrexate and glucocorticoid for >168 days; Combination 2, concurrent use of methotrexate, other conventional synthetic disease-modifying antirheumatic drugs, and glucocorticoid for >168 days; Combination 3, concurrent use of methotrexate and a biologic disease-modifying antirheumatic drug for >168 days.
