# Supplementary material for: Interpretable Machine Learning Framework for Predicting Major Adverse Cardiovascular Events in Rheumatoid Arthritis Using Electronic Health Records: Multicenter Cohort Study
Source: JMIR Form Res. 2026 Jun 5;10:e91790. doi: 10.2196/91790 (PMC13240640; doi:10.2196/91790)
Supplement: Multimedia Appendix 3 [file formative-v10-e91790-s003.docx]

Multimedia Appendix 3. Univariate and multivariate Cox proportional hazards regression analyses for predictors of major adverse cardiovascular events in patients with rheumatoid arthritis

| Variable | Univariate Cox PH model | | | | Multivariate Cox PH model | | | |
| --- | --- | --- | --- | --- | --- | --- | --- | --- |
|  | HR | 95% CI lower | 95% CI upper | *P value* | aHR | 95% CI lower | 95% CI upper | *P value* |
| Sex | 1.1 | 0.82 | 1.47 | .52 | 1.18 | 0.88 | 1.59 | .28 |
| Age | 1.05 | 1.04 | 1.06 | <.001 | 1.04 | 1.03 | 1.06 | <.001 |
| Diabetes mellitus | 1.86 | 1.35 | 2.57 | <.001 | 1.29 | 0.89 | 1.87 | .18 |
| Hypertension | 1.95 | 1.51 | 2.53 | <.001 | 1.38 | 1.03 | 1.85 | .03 |
| Hyperlipidemia | 1.35 | 1.04 | 1.75 | .03 | 1.21 | 0.91 | 1.62 | .19 |
| COPD | 1.86 | 1.39 | 2.49 | <.001 | 1.25 | 0.91 | 1.72 | .16 |
| MC | 1.9 | 0.71 | 5.11 | .20 | 1.14 | 0.37 | 3.47 | .82 |
| ILD | 3.16 | 1.41 | 7.1 | .01 | 1.56 | 0.68 | 3.61 | .30 |
| Cancer | 1.12 | 0.65 | 1.92 | .68 | 0.78 | 0.44 | 1.35 | .37 |
| Rheumatoid factor | 1 | 1 | 1 | .01 | 1 | 1 | 1 | .003 |
| C-reactive protein | 1 | 0.96 | 1.05 | .99 | 0.96 | 0.91 | 1.01 | .12 |
| ESR | 1.01 | 1 | 1.02 | <.001 | 1 | 1 | 1.01 | .40 |
| AST | 1 | 1 | 1.01 | .59 | 1 | 0.99 | 1.01 | .99 |
| ALT | 1 | 1 | 1.01 | .72 | 1 | 0.99 | 1.01 | .85 |
| Creatinine | 1.05 | 1.03 | 1.07 | <.001 | 1.04 | 1.01 | 1.07 | .004 |
| bDMARDs | 1 | 1 | 1 | <.001 | 1 | 1 | 1 | .07 |
| csDMARDs | 1 | 1 | 1 | <.001 | 1 | 1 | 1 | <.001 |
| Glucocorticoids | 1 | 1 | 1 | <.001 | 1 | 1 | 1 | .09 |
| Lipid-lowering agents | 1 | 1 | 1 | .05 | 1 | 1 | 1 | .01 |
| Antidiabetic agents | 1 | 1 | 1 | .91 | 1 | 1 | 1 | .84 |
| Medication combination^a^ | 0.73 | 0.63 | 0.85 | <.001 | 1.18 | 0.98 | 1.41 | .09 |

**Note:** PH, proportional hazards; HR, hazard ratio; aHR, adjusted hazard ratio; CI, confidence interval; COPD, chronic obstructive pulmonary disease; MC, multiple comorbidities, defined as concurrent diabetes, hypertension, hyperlipidemia, and chronic obstructive pulmonary disease; ILD, interstitial lung disease; ESR, erythrocyte sedimentation rate; AST, aspartate aminotransferase; ALT, alanine aminotransferase; bDMARDs, biologic disease-modifying antirheumatic drugs; csDMARDs, conventional synthetic disease-modifying antirheumatic drugs; ^a^Patients were classified into four medication combination groups: (1) those not meeting any of the following criteria; (2) concurrent use of methotrexate and glucocorticoids for more than 168 days; (3) concurrent use of methotrexate, other conventional synthetic disease-modifying antirheumatic drugs (csDMARDs), and glucocorticoids for more than 168 days; and (4) concurrent use of methotrexate and biologic disease-modifying antirheumatic drugs (bDMARDs) for more than 168 days. The first group served as the reference category in the Cox PH regression analysis.
