# Supplementary figures and images for: Interpretable Machine Learning Framework for Predicting Major Adverse Cardiovascular Events in Rheumatoid Arthritis Using Electronic Health Records: Multicenter Cohort Study
Source: JMIR Form Res. 2026 Jun 5;10:e91790. doi: 10.2196/91790 (PMC13240640; doi:10.2196/91790)

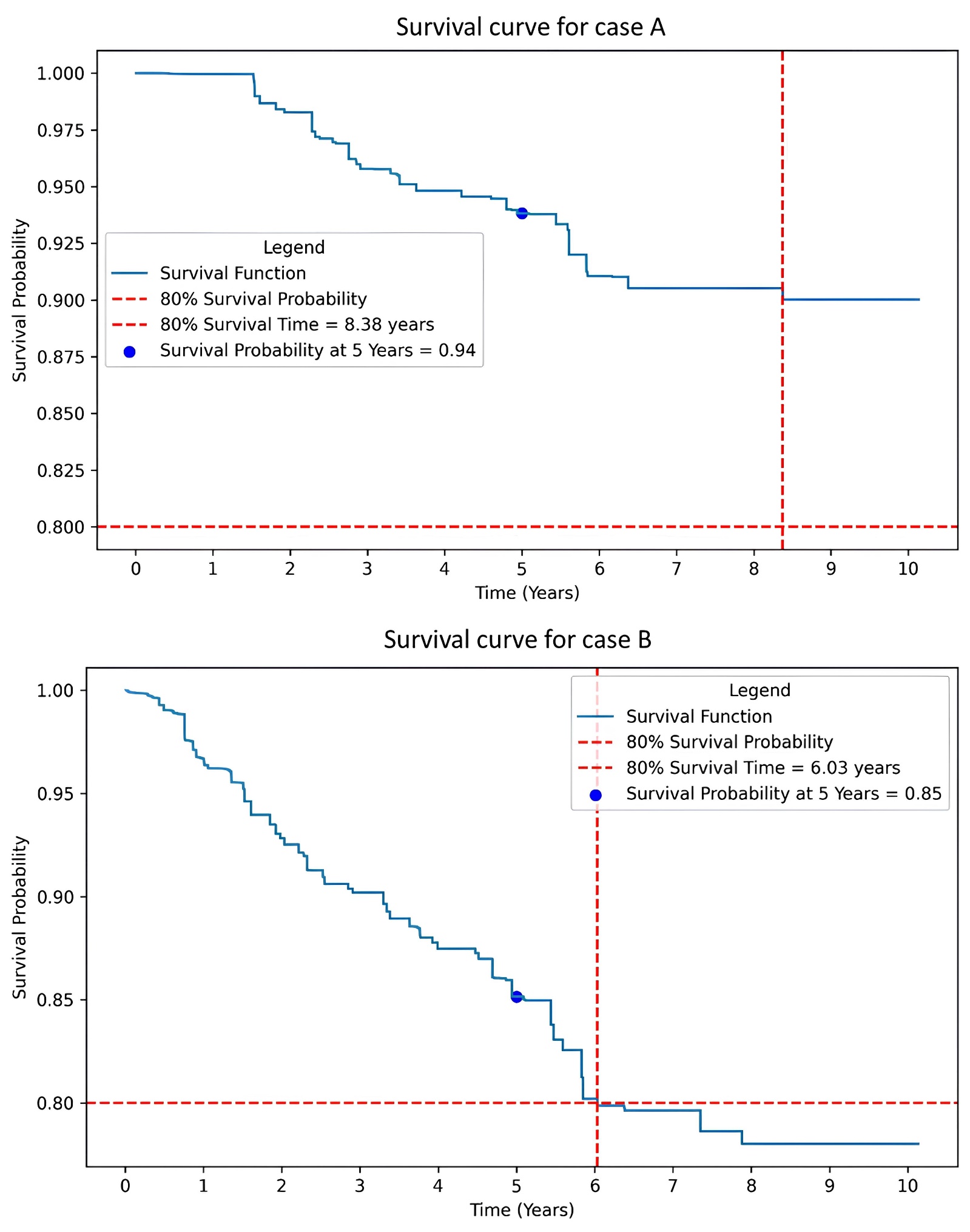


Multimedia Appendix 4. Kaplan-Meier Survival Curves for Case A and B

Supplement: Multimedia Appendix 4 [file formative-v10-e91790-s004.docx]
